# Prenatal exposure to glucocorticoids and the prevalence of overweight or obesity in childhood

**DOI:** 10.1530/EJE-21-0846

**Published:** 2022-02-01

**Authors:** Kristina Laugesen, Henrik Toft Sørensen, Jens Otto L Jorgensen, Irene Petersen

**Affiliations:** 1 Department of Clinical Epidemiology, Aarhus University Hospital, Aarhus, Denmark; 2 Department of Clinical Medicine, Aarhus University, Aarhus, Denmark; 3 Department of Endocrinology and Internal Medicine, Aarhus University Hospital, Aarhus, Denmark; 4 Department of Primary Care and Population Health, University College London, London, UK

## Abstract

**Objective:**

Prenatal exposure to excess cortisol can affect postnatal metabolic health by epigenetic mechanisms. We aimed to investigate if prenatal exposure to pharmacological glucocorticoids increases the risk of overweight/obesity in childhood.

**Design:**

A nationwide population registry-based cohort study.

**Methods:**

We identified 383 877 children born in Denmark (2007–2012), who underwent routine anthropometric evaluation at 5–8 years of age. Prenatal exposure to glucocorticoids was divided into systemic and topical glucocorticoids, cumulative systemic dose, and use by trimester. The comparison cohort included children without exposure, born to maternal never-users. Negative control exposures were used to investigate confounding from an underlying disease or unmeasured characteristics. Such exposures included children without glucocorticoid exposure born to maternal users of non-steroidal anti-inflammatory drugs or immunotherapy during pregnancy, maternal former users of glucocorticoids, or paternal users of glucocorticoids during the pregnancy of their partner. We estimated sex-stratified adjusted prevalence ratios (aPR) of overweight/obesity at 5–8 years of age, as epigenetic modifications have shown to be sex-specific.

**Results:**

In the study, 21 246 (11%) boys and 27 851 (15%) girls were overweight/obese at 5–8 years of age. Overall, neither systemic nor topical glucocorticoids were associated with overweight/obesity. In boys, high-dose systemic glucocorticoids was associated with higher prevalence of overweight/obesity vs the comparison cohort (aPR: 1.41 (95% CI: 1.07–1.86), prevalence: 16% vs 11%). Negative control exposures indicated robustness to confounding.

**Conclusion:**

Overweight/obesity might be an adverse effect of prenatal exposure to high-dose systemic glucocorticoids in boys. We found no association for neither prenatal exposure to lower doses of systemic nor topical glucocorticoids. These results merit clinical attention.

## Introduction

Overweight and obesity affect around 10–20% of children in Western countries (www.who.int/dietphysicalactivity/childhood/en/; Accessed August 6, 2021). Susceptibility is determined by genetic, epigenetic, and environmental risk factors (1). Evidence suggests that predisposition may be founded *in utero* via epigenetic changes and fetal programming (2). Consequently, it has been questioned if prenatal exposure to excess endogenous cortisol or synthetic glucocorticoids impacts obesity risk in an obesogenic postnatal environment (2, 3, 4, 5, 6). Cortisol is a major determinant of fetal development, and intrauterine bioactivity is tightly regulated by placental 11ß-hydroxysteroid dehydrogenase 2 (11β-HSD2) (7). Synthetic prednisolone is also a substrate for 11b-HSD2, but enzymatic saturation after high-dose or long-term treatment can lead to greater placental bypass. Further, certain synthetic glucocorticoids are not substrates (7). The prenatal glucocorticoid environment affects fetal epigenetics and impacts postnatal physiology. Such effects include changes in the sensitivity of the hypothalamic–pituitary–adrenal (HPA) axis, decreased insulin sensitivity, and alterations in adipocyte biology (4, 5, 8, 9, 10, 11, 12). Numerous human studies have shown that prenatal exposure to excess cortisol (stress) modulates metabolic health and increases obesity risk later in life with observed odds ratios between 1.1 and 2.0 (2, 8, 9, 13, 14, 15, 16). Animal studies suggest similar effects for synthetic glucocorticoids (10). Pharmacological glucocorticoid use is prevalent in pregnant women with autoimmune or inflammatory diseases, but whether such use increases obesity risk in offspring remains unknown.

We conducted a nationwide population registry-based cohort study to investigate if prenatal exposure to pharmacological glucocorticoids was associated with increased prevalence of overweight/obesity at 5–8 years of age as well as BMI z-scores.

## Subjects and methods

### Setting

Denmark has approximately 65 000 births annually. The Danish healthcare system provides tax-supported health services to all residents, guaranteeing access to health care free of charge (17). A unique identity number is assigned to all Danish residents at birth or emigration. This number is used in all health care contacts and is a key identifier in all registries, which allows continuous population surveillance and accurate and unambiguous linkage of relevant registries at the individual level (18).

### Study cohort

**Figure 1:**
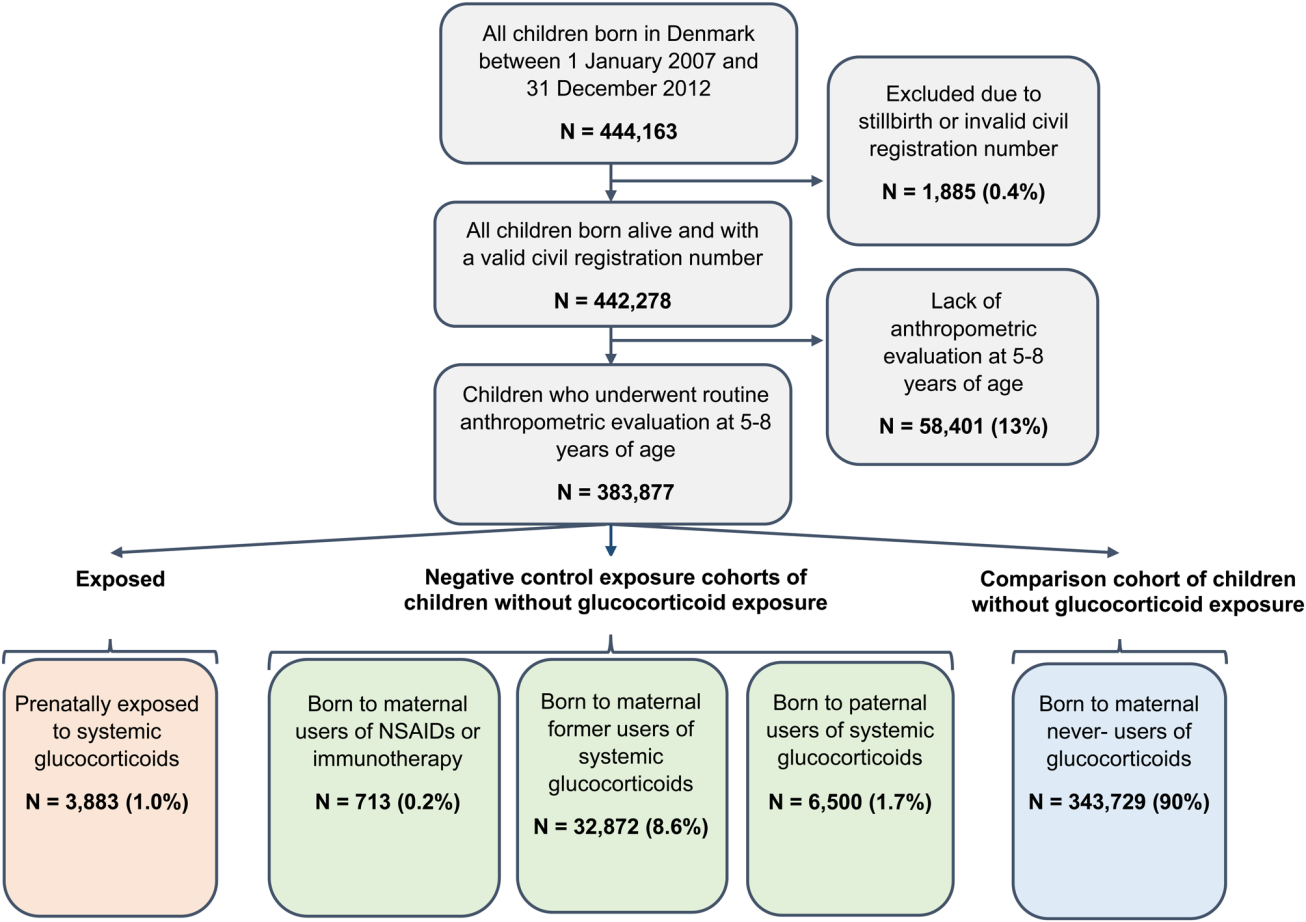
Study cohort. A full color version of this figure is available at https://doi.org/10.1530/EJE-21-0846.

We identified all children born alive in Denmark between January 1, 2007, and December 31, 2012, in the Danish Medical Birth Register, who underwent routine anthropometric evaluation at 5–8 years of age (at time of enrollment in primary school), and registered in the Danish National Children’s database (Fig. 1) (19). The Danish Medical Birth Register holds information on all births in Denmark since 1973 collected by the midwife or physician overseeing the delivery. Information collected includes the personal identity numbers of the infant, mother, and father, as well as information related to the pregnancy, the delivery, and infant characteristics (19). The Danish National Children’s database contains data on anthropometric variables (e.g. height and weight) of Danish school children from 2009 and onwards, measured by school nurses at enrolment in primary school, at check-ups, and at time of graduation from primary school. The database is complete from 2012 and onwards.

### Exposure and comparison cohorts

Using the Danish National Prescription Registry and the National Patient Registry, we defined prenatal glucocorticoid exposure as maternal redeemed prescriptions for glucocorticoids or a maternal hospital record of glucocorticoid treatment during pregnancy (20). The onset of pregnancy was defined as the first day of the last menstrual period, using the gestational age of the child at birth. We assessed both systemic and topical formulations, but they were analyzed separately (Supplementary Table 1, see section on supplementary materials given at the end of this article). Since 1995, the Prescription Registry has recorded pharmacy customers’ personal identity number, the classification code of the dispensed medication (using the Anatomical Therapeutic Chemical classification system of the World Health Organization (WHO)), date of dispensing, number of packages and tablets dispensed, and tablet strength among others (20). The Prescription Registry is considered complete and with valid information on medications dispensed in community pharmacies, but does not hold information on medications administered to hospitalized patients. Few drugs used for in-hospital treatment are recorded in the Patient Registry by procedure codes, including antenatal treatment with betamethasone for imminent preterm birth and other immunotherapies, such as biological treatments for rheumatic or inflammatory bowel disease (Supplementary Table 1) (21). In-hospital glucocorticoid treatment, besides antenatal betamethasone, is not captured in any Danish registry.

We defined exposure categories as follows:

- Children exposed any time during the pregnancy: if the mother redeemed one or more prescriptions or had a hospital record of glucocorticoid treatment any time from the start of pregnancy until delivery.

This overall category was further divided according to timing of systemic exposure into:

- Children exposed during the first trimester only*:*if the mother redeemed one or more prescriptions during the first 84 days of pregnancy, with no further prescriptions or hospital records of glucocorticoid treatment during the remainder of the pregnancy.
- Children exposed during the second trimester only:if the mother redeemed one or more prescriptions or had a hospital record of glucocorticoid treatment between day 85 and day 196, and no further prescriptions or hospital records during the remainder of the pregnancy.
- Children exposed during the third trimester only:if the mother redeemed one or more prescriptions or had a hospital record of glucocorticoid treatment between day 197 and the delivery date, with no prescriptions or hospital records during the first trimesters of the pregnancy.
- Children exposed during multiple trimesters:if the mother redeemed prescriptions or had a hospital record in more than one trimester.

We further assessed the cumulative systemic glucocorticoid dose expressed in prednisolone-equivalents (peq), calculated as the total number of tablets/injections dispensed during pregnancy multiplied by the strength of the tablets/injections and the peq conversion factor (Supplementary Table 2). This calculation led to discrete values of cumulative systemic exposure dose (Supplementary Table 3). Based on the exposure distribution, we categorized cumulative peq dose as follows: <250, 250–499, and ≥500 mg. High-dose glucocorticoid exposure was defined as cumulative exposure peq dose ≥500 mg.

The comparison cohort consisted of children without prenatal exposure born to maternal never-users of glucocorticoids.

### Negative control exposure cohorts

We established four negative control exposure cohorts of children without prenatal exposure to glucocorticoids defined as following:

- Children without prenatal glucocorticoid exposure but with prenatal non-steroidal anti-inflammatory drugs (NSAID) or immunotherapy exposure: if the mother redeemed at least one prescription for these agents or was treated in hospital settings with these agents any time during pregnancy and had no glucocorticoid use during pregnancy.

Children without prenatal glucocorticoid exposure born to maternal former users divided into:

- Children whose mothers used glucocorticoids >6–24 months before pregnancy:the mother redeemed her most recent prescription 6*–*24 months before the start of pregnancy and had no prescriptions or hospital records from 6 months before the start of pregnancy until delivery.
- Children whose mothers used glucocorticoids >24 months before pregnancy:the mother redeemed her most recent prescription >24 months before the start of pregnancy and had no prescriptions or hospital records from 24 months before the start of pregnancy until delivery.

Due to uncertainty of exposure status, children whose mothers used glucocorticoids 0*–*6 months before pregnancy were not included in the analyses. The oocyte may have been exposed to glucocorticoids in the periconceptional critical window of development, and it is further ambiguous if the mothers used glucocorticoids at the beginning of the pregnancy or not.

- Children without prenatal glucocorticoid exposure but with prenatal paternal use of systemic glucocorticoids:if the father redeemed one or more prescriptions for systemic glucocorticoids during the pregnancy of his partner, but never before the start of pregnancy (i.e. only new users). The ‘new use’ criteria were applied to ensure that these children served solely as a negative control exposure cohort since some evidence suggests that fetal glucocorticoid programming may be transmitted via paternal germline lineages (22). The paternal negative control exposure cohort aimed to identify potential genetic or family-related confounding.

The purpose of the negative control cohorts was to investigate potential confounding from factors such as underlying disease, underlying disease severity, or unmeasured characteristics in the main analysis (assessing the exposure and comparison cohorts). For example, maternal glucocorticoid users during pregnancy, maternal former users of glucocorticoids, and maternal users of NSAIDs or immunotherapy during pregnancy may share common traits, such as treatment indication. We expected no causal associations when comparing the negative control exposure cohorts vs the comparison cohort. Hence, findings of an association would indicate confounding in our main analysis.

### BMI, overweight, and obesity

We used the Danish National Children’s Database to obtain data on height and weight of children in our study cohort at 5–8 years of age. BMI was calculated by dividing an individual’s weight in kilograms by the square of height in meters. The Extended International (IOTF) BMI cut-offs were used to define overweight or obesity (BMI ≥ IOTF-25) www.worldobesity.org/about/about-obesity/obesity-classification; Accessed August 6, 2021) (23). The WHO 2007 reference data and macro were used to obtain BMI z-scores (www.who.int/toolkits/growth-reference-data-for-5to19-years/application-tools; Accessed August 6 2021).

### Descriptive characteristics and covariates

For descriptive purposes only (i.e. not confounding control), we assessed the following delivery and birth characteristics, as captured in the Danish Medical Birth Registry: sex of the child, birth order (1 and ≥2), gestational age (<28, 28–31, 32–36, and ≥37 weeks), birth weight, small for gestational age, Apgar score after 5 min (<7 and 7–10), and cesarean section status.

We used directed acyclic graphs (DAGs) to identify confounding based on a priori knowledge (Supplementary Fig. 1). Potential confounding variables included maternal age at birth, maternal BMI at start of pregnancy, smoking (yes/no), potential treatment indications (obstructive pulmonary disease including asthma or chronic obstructive pulmonary disease, inflammatory bowel disease, rheumatic disease, renal disease, and skin disease), maternal comorbidities such as maternal type I or II diabetes, polycystic ovarian syndrome, psychiatric illnesses, and infections or antibiotic use during pregnancy. Treatment indications were identified based on hospital records (in- and outpatient). As a measure of disease severity, we assessed the number of hospital contacts for each treatment indication within 2 years before birth (0, 1–4, and >4). Comorbidities were detected by either relevant drug prescribing or hospital records (in- and outpatient) (Supplementary Table 4). The highest maternal educational level at the birth of the child was obtained from the Danish social and demographic registries. The highest educational level was classified as low (primary and lower secondary education), medium (upper secondary education or professional degree), and high (university education at the bachelor’s degree level or higher).

### Statistical analysis

We described the study cohort according to exposure status, birth characteristics, and maternal characteristics. As fetal programming may be sex-dependent (4, 13, 24), we stratified all analyses by sex.

We reported the prevalence of overweight or obesity (combined) according to prenatal exposure status. Using robust Poisson regression with generalized estimating equations (family Poisson and link as log), we estimated crude and adjusted prevalence ratios (aPRs), comparing the exposure cohort with the comparison cohort. We further examined the associations by trimester and cumulative systemic glucocorticoid dose. Likewise, the negative control exposure cohorts were compared with the comparison cohort. Due to few outcomes, we were unable to assess overweight and obesity as two separate outcomes. We calculated BMI z-scores using the WHO 2007 reference data and macro and verified that these were normally distributed. We estimated crude and adjusted differences in mean BMI z-scores, comparing the exposure cohort with the comparison cohort, using generalized estimating equations with robust standard errors (family gauss and link as identity). Again, we computed mean differences by trimester and cumulative systemic glucocorticoid dose. The adjusted models were adjusted for confounding factors as described above (Supplementary Fig. 1).

In a supplemental analysis, we investigated the association between maternal characteristics and glucocorticoid exposure as well as offspring overweight/obesity. Further, to account for unmeasurable in-hospital glucocorticoid treatment, we conducted a sensitivity analysis and excluded mothers with potential in-hospital treatment during pregnancy. These mothers were defined as being hospitalized with a glucocorticoid treatment indication during pregnancy (Supplementary Table 4).

Statistical analyses were conducted using Stata version 16.

## Results

### Study cohort

We identified 442 278 children born alive in Denmark between 2007 and 2012. Of these, 383 877 (87%) underwent routine anthropometric evaluation at 5–8 years of age (Fig. 1). Children with and without anthropometric evaluation did not differ with regard to exposure frequency (Supplementary Table 5). In our study cohort, 3883 (1.0%) were prenatally exposed to systemic glucocorticoids (median cumulative dose 200 mg peq), and 43 131 (11%) were prenatally exposed to topical glucocorticoids only (Table 1). The most frequent hospital-based treatment indications were asthma (11%), inflammatory bowel disease (6.4%), and rheumatic disease (6.0%). The negative control exposure cohorts consisted of children without glucocorticoid exposure, of whom 32 872 (8.6%) were born to maternal former users of systemic glucocorticoids, 713 (0.2%) were born to maternal users of NSAIDs or immunotherapies during pregnancy, and 6500 (1.7%) were born to paternal users of systemic glucocorticoids (Fig. 1).

**Table 1:** Prenatal exposure. Data are presented as *n* (%).

Exposure	All children	Boys	Girls
All	383 877 (100)	195 689 (100)	188 188 (100)
Exposed to systemic glucocorticoids	3883 (1.0)	2011 (1.0)	1872 (1.0)
Timing of exposure			
First trimester only	1179 (0.3)	601 (0.3)	578 (0.3)
Second trimester only	638 (0.2)	341 (0.2)	287 (0.2)
Third trimester only	1709 (0.5)	884 (0.5)	825 (0.4)
Multiple-trimester exposure	357 (0.09)	185 (0.09)	172 (0.09)
Generic systemic glucocorticoid type			
Prednisolone only	1498 (0.4)	778 (0.4)	720 (0.4)
Prednisone only	273 (0.07)	132 (0.07)	141 (0.07)
Methylprednisolone only	149 (0.04)	75 (0.04)	74 (0.04)
Betamethasone only	1842 (0.5)	986 (0.5)	874 (0.5)
Dexamethasone only	0	0	0
Hydrocortisone only	42 (0.01)	18 (0.01)	24 (0.01)
Triamcinolone only	NA	NA	NA
Multiple types	68 (0.02)	35 (0.02)	33 (0.02)
Cumulative systemic glucocorticoid dose in prednisolone-equivalents^a^, median (IQR)	200 mg (200–500 mg)	200 mg (200–500 mg)	200 mg (200–500 mg)
High-dose systemic exposure (≥500 mg peq)	593 (0.2)	309 (0.2)	284 (0.2)
Exposed to topical glucocorticoids only	43 131 (11)	22 261 (11)	20 970 (11)
Former maternal systemic use (<6 months since most recent prescription)^b^	3393 (0.9)	1727 (0.9)	1666 (0.9)
Former maternal systemic use (6–24 months since most recent prescription)	6931 (1.8)	3500 (1.8)	3431 (1.8)
Former maternal systemic use (>24 months since most recent prescription)	25 941 (6.8)	13 178 (6.7)	12 763 (6.8)
Maternal use of NSAIDs or immunotherapy during pregnancy	713 (0.2)	367 (0.2)	346 (0.2)
Paternal systemic glucocorticoid use	6500 (1.7)	3318 (1.7)	3182 (1.7)

^a^The cumulative systemic glucocorticoid dose in prednisolone-equivalents was calculated by multiplying the number of pills/injections, dose per pill/injection, and prednisolone conversion factor for the cumulative prescriptions during pregnancy. ^b^Not included in the analyses due to uncertainty in exposure status.

IQR, interquartile range. NA, not applicable due to data legislation. NSAIDs, non-steroidal anti-inflammatory drugs.

### Characteristics

Children prenatally exposed to systemic glucocorticoids were more likely to be small for gestational age at birth (14%) vs children in the comparison cohort (9.5%) or negative control exposure cohorts (8.4–9.4%). Likewise, prenatally exposed children were less likely to be born at term (59% vs 93% in the comparison cohort and 91–94% in the negative control exposure cohorts) (Table 2).

**Table 2:** Birth and infant as well as parental characteristics of 383,877 children according to prenatal exposure. Data are presented as *n* (%) or as median (IQR). Children whose mothers used glucocorticoids 0–6 months before pregnancy were not included due to uncertainty in exposure status. Children prenatally exposed to systemic glucocorticoids were more likely to be SGA at birth and have an Apgar score <7 vs children in the comparison cohort or negative control exposure cohorts. Moreover, prenatally exposed children were less likely to be born at term. Highest educational level at birth: low (primary and lower secondary education), medium (upper secondary education or professional degree), and high (university education at bachelor’s degree level or higher. Children whose mothers used glucocorticoids 0–6 months before pregnancy were not included due to uncertainty in exposure status. Infections/use of antibiotics during pregnancy as well as apsychiatric disease were more frequent among both maternal systemic glucocorticoid users and among mothers in the negative control exposure cohorts compared to maternal never-users. Further, the frequency of diabetes was higher among maternal systemic glucocorticoid users compared to all other cohorts.

	Prenatally exposed to systemic glucocorticoids	Children without prenatal exposure				
		Comparison cohort	Negative control exposure cohorts			
		Maternal never-use	Maternal former use (>24 months before pregnancy)	**Maternal former use (6–24 months before pregnancy)**	Maternal use of NSAIDs or immunotherapy during pregnancy	Paternal use of systemic glucocorticoids
Birth and infant characteristics						
All births	3883 (100)	343 729 (100)	25 941 (100)	6931 (100)	713 (100)	6500 (100)
Sex						
Male	2011 (52)	175 273 (51)	13 178 (51)	3500 (51)	367 (51)	3318 (51)
Gestational age, weeks						
<28	251 (6.5)	446 (0.1)	39 (0.2)	19 (0.3)	0 (0)	7 (0.1)
28–31	331 (8.5)	2,164 (0.6)	193 (0.7)	37 (0.5)	NA	34 (0.5)
32–36	1016 (26)	19 840 (5.7)	1742 (6.7)	516 (7.4)	59 (8.3)	327 (5.0)
≥37	2285 (59)	321 279 (93)	23 967 (92)	6359 (92)	652 (91)	6132 (94)
Birth weight in g	2940 (2060-3486)	3510 (3160-3850)	3500 (3138-3850)	3480 (3110-3830)	3438 (3100-3774)	3522 (3174-3858)
Missing	24 (0.6)	6213 (1.8)	286 (1.1)	56 (0.8)	8 (1.1)	91 (1.4)
SGA	558 (14)	32 477 (9.5)	2404 (9.3)	646 (9.3)	60 (8.4)	613 (9.4)
Apgar score after 5 min						
<7	88 (2.3)	2072 (0.6)	166 (0.6)	58 (0.8)	6 (0.8)	49 (0.8)
7–10	3779 (97)	335 640 (98)	25 504 (98)	6823 (98)	699 (98)	6362 (98)
Missing	16 (0.4)	6017 (1.8)	271 (1.0)	50 (0.7)	8 (1.1)	89 (1.4)
Cesarean section	724 (32)	69 203 (20)	6353 (24)	1889 (27)	248 (35)	1368 (21)
Multiple birth	893 (23)	13 297 (3.9)	1283 (5.0)	308 (4.4)	9 (1.3)	180 (2.8)
Parental characteristics						
All births	3883 (100)	343 729 (100)	25 941 (100)	6931 (100)	713 (100)	6500 (100)
Maternal characteristics						
Age (years) at birth	32 (29–36)	30 (27–34)	31 (28–34)	31 (28–34)	30 (28–34)	30 (27–34)
Parity						
1	2156 (55)	151 745 (44)	10 764 (42)	2937 (42)	364 (51)	2687 (41)
≥2	1 651 (43)	181 095 (53)	14 601 (56)	3845 (55)	334 (47)	3647 (56)
Missing	76 (2.0)	10 889 (3.2)	576 (2.2)	149 (2.2)	15 (2.1)	166 (2.6)
Highest educational level						
Low	567 (15)	56 222 (16)	3228 (12)	1158 (17)	91 (13)	1119 (17)
Medium	1457 (38)	139 740 (41)	11 408 (44)	3116 (46)	313 (44)	2970 (46)
High	1764 (45)	139 740 (41)	11 001 (42)	2493 (36)	301 (42)	2288 (35)
Missing	95 (2.5)	8550 (2.5)	304 (1.2)	114 (1.6)	8 (1.1)	123 (1.9)
BMI (kg/m^2^) at start of pregnancy	23 (21–26)	23 (21–26)	24 (21–27)	24 (21–28)	23 (21–27)	23 (21–27)
<18.5	171 (4.4)	14 355 (4.2)	846 (3.3)	238 (3.4)	23 (3.2)	230 (3.5)
18.5–24	2360 (61)	202 516 (59)	14 402 (56)	3640 (53)	407 (57)	3721 (57)
25–29	726 (19)	66 828 (19)	5598 (22)	1577 (23)	146 (20)	1328 (13)
≥30	429 (11)	38 615 (11)	3849 (15)	1145 (17)	111 (16)	858 (13)
Missing	187 (4.8)	21 415 (6.2)	1246 (4.8)	331 (4.8)	26 (3.7)	363 (5.6)
Smoking during pregnancy						
Yes	430 (11)	44 235 (13)	3193 (12)	984 (14)	109 (15)	891 (14)
Missing	180 (4.6)	12,479 (3.6)	807 (3.1)	213 (3.1)	14 (2.0)	217 (3.3)
Hospital diagnosed treatment indications						
Obstructive pulmonary disease	443 (11)	11 323 (3.3)	3401 (13)	939 (14)	41 (5.8)	269 (4.1)
Inflammatory bowel disease	247 (6.4)	2563 (0.8)	1233 (4.8)	354 (5.1)	293 (41)	74 (1.1)
Rheumatic disease	231 (6.0)	2038 (0.6)	522 (2.0)	180 (2.6)	119 (17)	54 (0.8)
Renal disease	76 (2.0)	1394 (0.4)	229 (0.9)	55 (0.8)	12 (1.7)	37 (0.6)
Skin disease	93 (2.4)	5563 (1.6)	555 (2.2)	174 (2.5)	26 (3.7)	102 (1.6)
Comorbidities						
Diabetes (type 1, 2, gestational)	445 (11)	18 158 (5.3)	1845 (7.1)	507 (7.3)	38 (5.3)	358 (5.5)
Gestational diabetes during pregnancy	239 (6.2)	9560 (2.8)	915 (3.5)	273 (3.9)	27 (3.8)	199 (3.1)
Infections during pregnancy	1526 (39)	99 636 (29)	8397 (32)	2636 (38)	295 (41)	2419 (37)
Polycystic ovarian syndrome	76 (2.0)	4201 (1.2)	455 (1.8)	121 (1.8)	8 (1.1)	105 (1.6)
Psychiatric illness	1308 (34)	83485 (24)	8908 (34)	2579 (37)	234 (33)	1811 (28)
Mood or anxiety disorders	752 (19)	49104 (14)	5207 (20)	1608 (23)	128 (18)	1087 (17)
Substance use disorders	139 (3.6)	10 786 (3.1)	870 (3.4)	294 (4.2)	30 (4.2)	211 (3.3)
Use of antipsychotics during pregnancy	18 (0.46)	713 (0.21)	56 (0.22)	35 (0.50)	6 (0.84)	18 (0.28)

IQR, interquartile range. NSAIDs, non-steroidal anti-inflammatory drugs. SGA, small for gestational age defined as birth weight < the 10th percentile for infants of the same gestational age, sex, and birth year.

Maternal BMI was similar in all cohorts (median 23–24) (Table 2). Infections/use of antibiotics during pregnancy (32–41% vs 29%) as well as psychiatric disease (28–37% vs 24%) were more frequent among both maternal systemic glucocorticoid users and among mothers in the negative control exposure cohorts compared to maternal never-users. Further, the frequency of diabetes was higher among maternal systemic glucocorticoid users (11%) compared to all other cohorts (5.3–7.3%) (Table 2). The associations shown in Supplementary 6 confirmed our predefined confounding (Supplementary Fig. 1). Almost all selected covariates, including maternal age, educational level, BMI, smoking status, and comorbidities, were associated with overweight/obesity in the child as well as prenatal glucocorticoid exposure. The strongest predictors of overweight/obesity in the child were maternal BMI, diabetes, and antipsychotic drug use.

### Overweight/obesity

**Figure 2:**
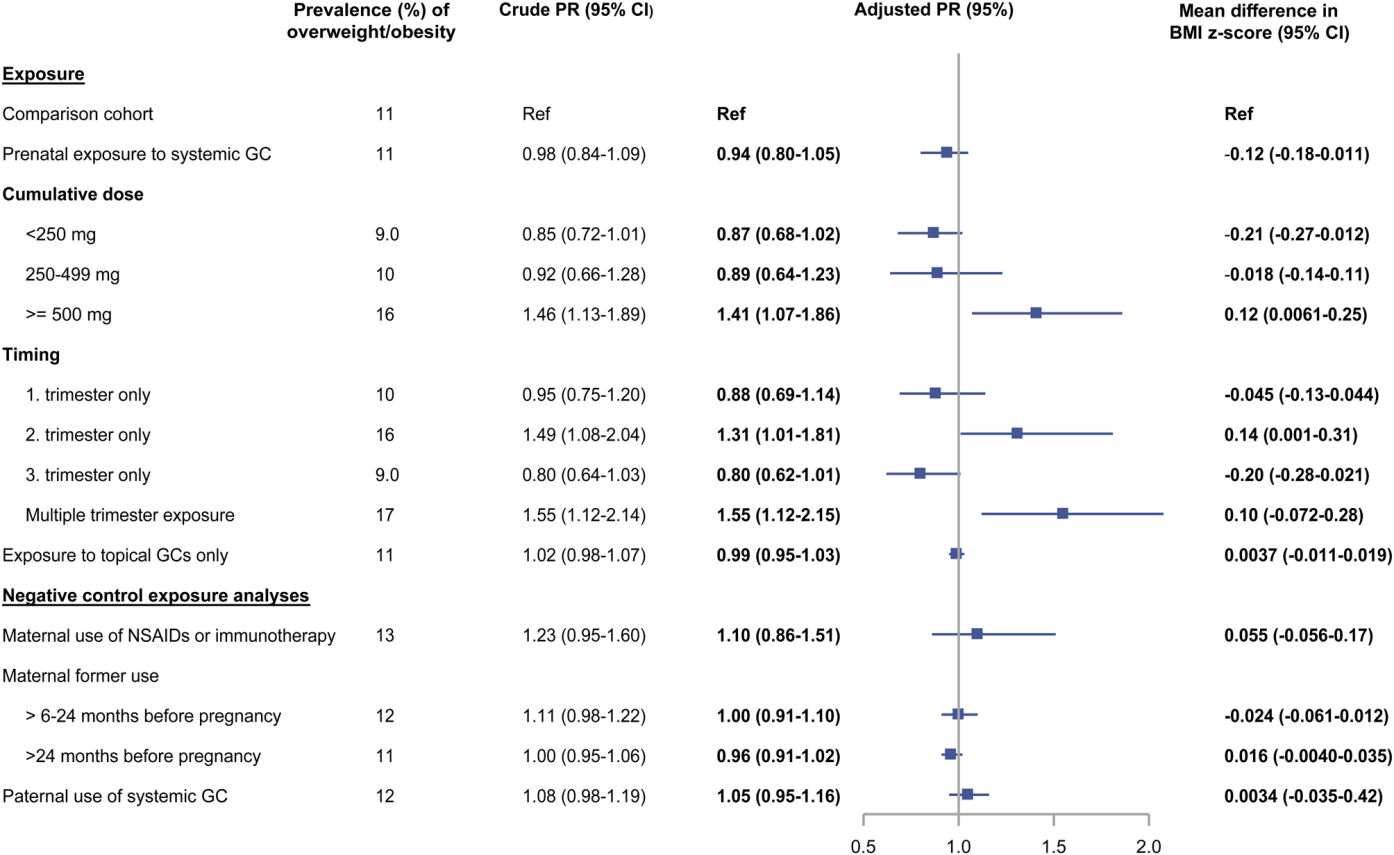
The association between prenatal exposure to glucocorticoids and the prevalence of overweight or obesity and BMI z-scores in 195 689 boys at 5–8 years of age. Adjusted for maternal age at birth (restricted cubic spline with three knots), maternal BMI at start of pregnancy (restricted cubic spline with three knots), smoking (yes/no), treatment indications, number of hospital contacts with treatment indications within 2 years prior to birth, maternal type I, II, or gestational diabetes, polycystic ovarian syndrome, psychiatric illnesses, and infections or antibiotic use during pregnancy. GC, glucocorticoids; NSAIDs, non-steroidal anti-inflammatory drugs; PR, prevalence ratio. A full color version of this figure is available at https://doi.org/10.1530/EJE-21-0846.

**Figure 3:**
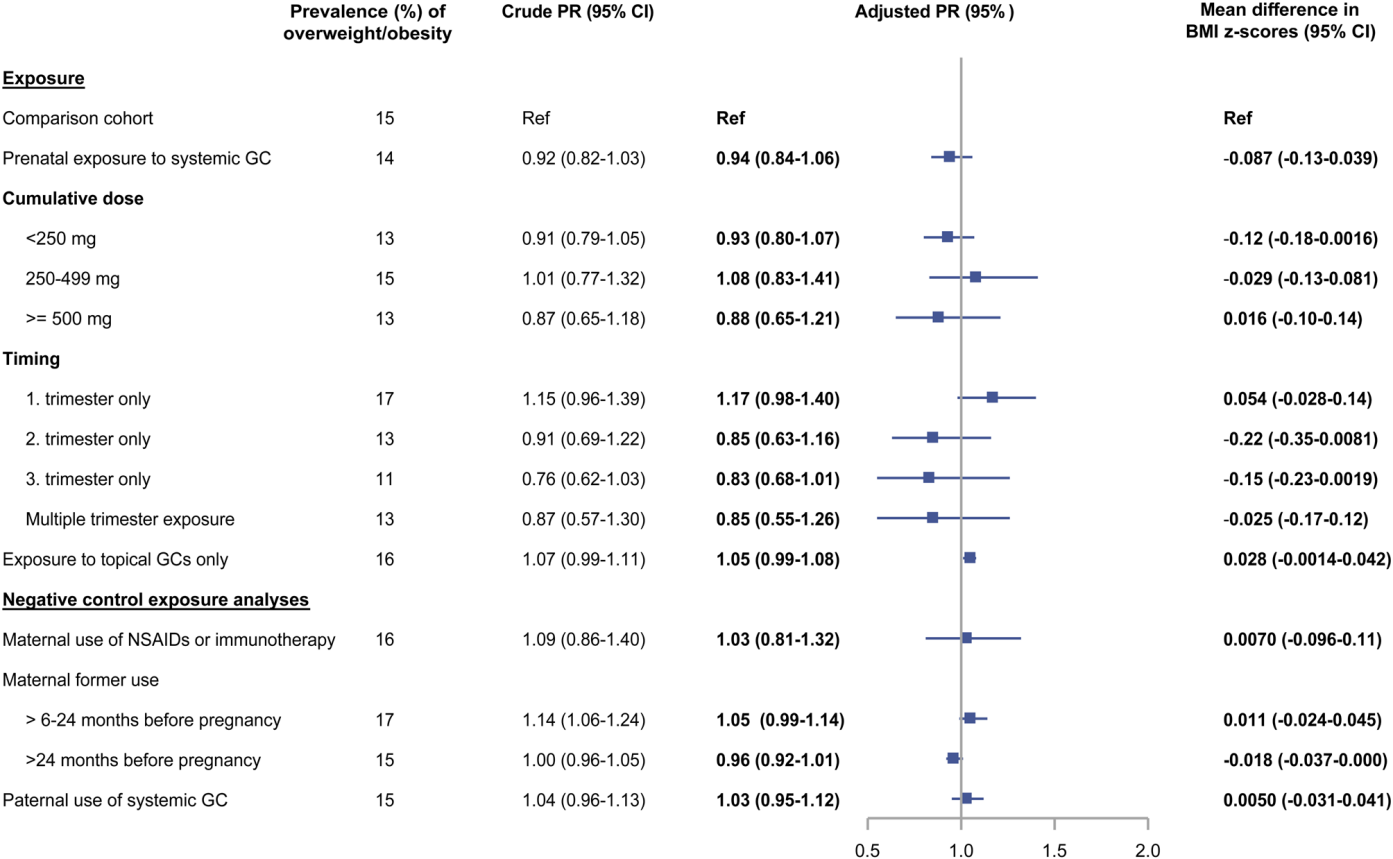
The association between prenatal exposure to glucocorticoids and the prevalence of overweight or obesity and BMI z-scores in 188 188 girls at 5–8 years of age. Adjusted for maternal age at birth (restricted cubic spline with three knots), maternal BMI at start of pregnancy (restricted cubic spline with three knots), smoking (yes/no), treatment indications, number of hospital contacts with treatment indications within two years prior to birth, maternal type I, II, or gestational diabetes, polycystic ovarian syndrome, psychiatric illnesses, and infections or antibiotic use during pregnancy. GC, glucocorticoids; NSAIDs, non-steroidal anti-inflammatory drugs; PR, prevalence ratio. A full colour version of this figure is available at https://doi.org/10.1530/EJE-21-0846.

A total of 21 246 (11%) boys and 27 851 (15%) girls were overweight/obese at 5–8 years of age. Overall, neither systemic nor topical glucocorticoids were associated with overweight/obesity in childhood (Figs 2 and 3). In boys, prenatal exposure to high-dose systemic glucocorticoids was associated with higher prevalence of overweight/obesity vs the comparison cohort at age 5-8 years (aPR 1.41 (95% CI: 1.07–1.86), prevalence 16% vs 11%) (Fig. 2). Difference in mean BMI z-scores was 0.12 (95% CI: 0.0061–0.25) for high-dose systemic exposure vs the comparison cohort (Fig. 2). We observed rather imprecise estimates for trimester exposure but found the strongest association for second trimester exposure (Fig. 2). No association between glucocorticoid exposure and overweight/obesity was observed in girls across either measures (Fig. 3). All negative control exposure analyses yielded null associations (Figs 2 and  3). Further, when we excluded women with potential unmeasured in-hospital glucocorticoid treatment during pregnancy, the results were almost unchanged (Supplementary Table 7).

## Discussion

In boys, prenatal exposure to cumulative high-dose systemic glucocorticoids was associated with a 1.4-fold increased prevalence of overweight or obesity at 5–8 years of age compared to children without exposure. We found no association for neither prenatal exposure to lower doses of systemic nor topical glucocorticoids.

Our study was conducted in a universal free tax-supported healthcare system and nationwide coverage of registries and databases enabled us to assess BMI in a large proportion of Danish children (17). Around 13% of eligible children did not have a routine anthropometric evaluation at 5–8 years of age. Children with and without anthropometric evaluation did not differ in terms of exposure frequency, hence selection bias is unlikely to explain our findings. As a proxy for maternal glucocorticoid use, we used prescription redemption. The Prescription Registry is considered complete for medications dispensed in community pharmacies but does not hold information on in-hospital medication use (20). In-hospital glucocorticoid treatment, besides antenatal treatment with betamethasone for imminent preterm birth, is not captured in the Danish registries. A small proportion of maternal glucocorticoid users could therefore have been misclassified as non-users. Further, non-users may have been misclassified as users if they redeemed a prescription but did not adhere to the treatment. Exposure misclassification was likely independent of the outcome and thus non-differential. Non-differential misclassification might lead to bias toward the null for our binary comparisons (e.g. any systemic glucocorticoid use vs the comparison cohort) and thus attenuate the prevalence ratios, but bias in an unpredictable direction for our non-binary comparisons (such as the dose-response analyses). We controlled for measured confounding, including treatment indications, comorbidity, educational level, and lifestyle (BMI, smoking). Further, our negative control exposure analyses confirmed the robustness of our findings in the context of shared confounding between the cohorts, such as maternal underlying disease, or other shared characteristics. The paternal negative control exposure cohort showed null results, indicating robustness to potential genetic or family-related confounding. Still, residual confounding from morbidity, lifestyle, or genetics cannot be entirely ruled out. We did not include information on breastfeeding of the baby or postnatal lifestyle. A former study, however, showed that diet and physical activity did not differ substantially between glucocorticoid users and non-users in women of reproductive age (25).

Mechanisms that underlie programming by prenatal excess glucocorticoid exposure include epigenetic changes, notably affecting the tissue-specific expression of the glucocorticoid receptor. This may permanently alter tissue glucocorticoid signaling and the sensitivity of the HPA axis to increased basal and stress-induced cortisol levels (4, 5, 8, 9, 10), diminished pancreatic beta-cell mass, decreased insulin sensitivity, and change in adipocyte biology (26). Further, some of the effects may be mediated through intrauterine growth restriction, which is a predictor of metabolic disease later in life (the Barker hypothesis) (2, 3, 27, 28) in accordance with which we observed a higher prevalence of small for gestational age in prenatally exposed children. Further, glucocorticoid treatment may induce hyperglycemia, which is associated with an increased risk of obesity in the offspring (29). Mediation analysis (e.g. small for gestational age or hyperglycemia) was out of the scope of this study and also not possible due to sparse data, but may be of interest for future studies. We observed an association in boys but not girls. Differences in vulnerability to glucocorticoid-induced fetal programming between sexes have been described earlier (4, 13, 24). The mechanism behind such difference is not fully understood but may be due to sex-specific placental responsivity (24). We observed rather imprecise estimates for trimester exposure but found the strongest association for second-trimester exposure, which aligns with a recent study, showing that overweight in offspring was associated with higher maternal saliva cortisol levels during the second trimester of pregnancy (9).

The implications of this study largely affect pregnant women with autoimmune or inflammatory diseases, for which clinicians may consider glucocorticoid-sparring strategies as an alternative. However, risks should be weighed against benefits, as an inadequately treated maternal disease may also affect both mother and fetus. Neither prenatal exposure to lower doses of systemic nor topical glucocorticoids was associated with overweight/obesity in childhood. Yet, overweight and obesity are potential adverse effects of prenatal exposure to high-dose systemic glucocorticoids in boys. Future research needs to elucidate causality and potential mechanism for our findings.

## Supplementary Material

Supplementary Figure 1

Supplementary Table 1. Anatomical Therapeutic Chemical (ATC) Classification codes and procedure codes for relevant drug use.

Supplementary Table 2. Equivalency of systemic glucocorticoids and corresponding prednisolone conversion factors.

Supplementary Table 3. Distribution of cumulative systemic glucocorticoid dose expressed in prednisolone- equivalents (peq).

Supplementary Table 4. Definition of covariates.

Supplementary Table 5. Comparison of exposure and baseline characteristics of children with and without anthropometric evaluation at 5-8 years of age.

Supplementary Table 6. The association between the baseline characteristics with overweight/obesity in offspring and maternal use of systemic glucocorticoids during pregnancy.

Supplementary Table 7. Sensitivity analysis excluding mothers with potential unmeasured in-hospital glucocorticoid treatment (defined as being hospitalised with a glucocorticoid treatment indication during pregnancy).

## Declaration of interest

All authors declare no conflicts of interest. The Department of Clinical Epidemiology, Aarhus University Hospital, receives funding for other studies from companies in the form of research grants to (and administered by) Aarhus University. None of these studies have any relation to the present study.

## Funding

This work was supported by a grant from Novo Nordisk http://dx.doi.org/10.13039/501100004191 Fonden (grant no. 058005). The funding source had no role in the design of the study; the collection, analysis, and interpretation of the data; or the decision to submit the finished manuscript for publication.

## Ethical approval

This study was approved by the Danish Data Protection Agency (Record number: 2016–051-000001, serial number 1804). According to Danish legislation, informed consent or approval from an ethics committee is not required for registry-based studies.

## Author contribution statement

All authors contributed to the study’s conception. K L wrote the initial manuscript and performed statistical analyses. K L had full access to all study data and takes responsibility for the integrity of the data and the accuracy of the data analysis. I P, H T S, and J O L J contributed to the interpretation of results and revised the manuscript critically. All authors approved the final manuscript. K L is the guarantor for the study.
