# Supplementary figures and images for: Prenatal exposure to glucocorticoids and the prevalence of overweight or obesity in childhood
Source: Eur J Endocrinol. 2022 Feb 1;186(4):429–40. doi: 10.1530/EJE-21-0846 (PMC8942335; doi:10.1530/EJE-21-0846)

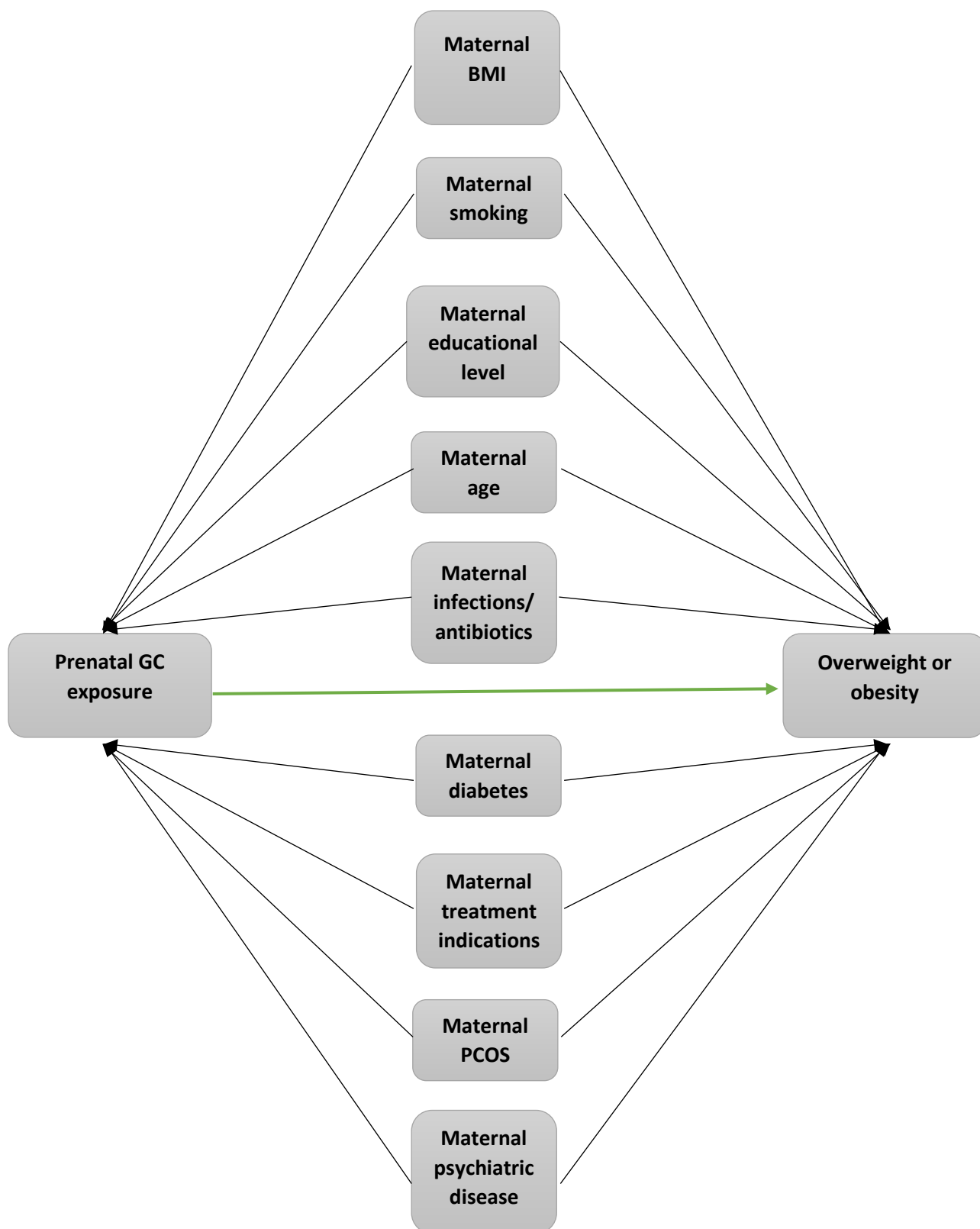

Supplement: Supplementary Figure 1 [file supplementary_figure_1.pdf]
