# Supplementary material for: Prenatal exposure to glucocorticoids and the prevalence of overweight or obesity in childhood
Source: Eur J Endocrinol. 2022 Feb 1;186(4):429–40. doi: 10.1530/EJE-21-0846 (PMC8942335; doi:10.1530/EJE-21-0846)
Supplement: Supplementary Table 1. Anatomical Therapeutic Chemical (ATC) Classification codes and procedure codes for relevant drug use. [file supplementary_table_1.pdf]

Supplementary Table 1. Anatomical Therapeutic Chemical (ATC) Classification codes and procedure codes for relevant drug use.

Medication type	ATC codes	Procedure codes
**Systemic glucocorticoids (oral or injectable)**		
Betamethasone	H02AB01	BBHF3
Dexamethasone	H02AB02	
Methylprednisolone	H02AB04	
Prednisolone	H02AB06	
Prednisone	H02AB07	
Triamcinolone	H02AB08	
Hydrocortisone	H02AB09	
**Inhaled glucocorticoids**		
Beclomethasone	R03BA01	
Budesonide	R03BA02	
Flunisolide	R03BA03	
Fluticasone	R03BA05	
Mometasone	R03BA07	
Ciclosonide	R03BA08	
**Glucocorticoids acting on the intestines**		
Hydrocortisone	A07EA01	
Budesonide	A07EA02<br>A07EA06	
Various topical glucocorticoids for haemorrhoids	C05AA	
**Topical glucocorticoids for skin conditions**	D07	
**Negative control exposure cohort**		
NSAIDS	M01A	
Immunotherapy	L04<br>P01BA02<br>A07EC01	BOHJ
