# Supplementary material for: Prenatal exposure to glucocorticoids and the prevalence of overweight or obesity in childhood
Source: Eur J Endocrinol. 2022 Feb 1;186(4):429–40. doi: 10.1530/EJE-21-0846 (PMC8942335; doi:10.1530/EJE-21-0846)
Supplement: Supplementary Table 2. Equivalency of systemic glucocorticoids and corresponding prednisolone conversion factors. [file supplementary_table_2.pdf]

Supplementary Table 2. Equivalency of systemic glucocorticoids and corresponding prednisolone conversion factors.

	Equivalent glucocorticoid dose	Prednisolone conversion factor
Hydrocortisone	20	0.25
Methylprednisolone	4	1.25
Prednisolone	5	1
Prednisone	5	1
Dexamethasone	0.75	6.67
Betamethasone	0.6	8.33
Triamcinolone	4	1.25

Cumulative dose calculation:

The cumulative prednisolone-equivalent dose was calculated by multiplying the number of pills/injections, dose per pill/injection, and prednisolone conversion factor for the drug of interest.
