# Supplementary material for: Prenatal exposure to glucocorticoids and the prevalence of overweight or obesity in childhood
Source: Eur J Endocrinol. 2022 Feb 1;186(4):429–40. doi: 10.1530/EJE-21-0846 (PMC8942335; doi:10.1530/EJE-21-0846)
Supplement: Supplementary Table 3. Distribution of cumulative systemic glucocorticoid dose expressed in prednisolone- equivalents (peq). [file supplementary_table_3.pdf]

Supplementary Table 3. Distribution of cumulative systemic glucocorticoid dose expressed in prednisolone- equivalents (peq).

Cumulative systemic glucocorticoid dose (peq)	Number of prenatally exposed children (%)
All doses	3,3883 (100)
50	64 (1.7)
58	65 (1.7)
100	66 (1.7)
116	104 (2.7)
125	287 (7.4)
200	1,640 (42)
250	413 (11)
375	29 (0.8)
500	564 (15)
750	32 (0.8)
1000	123 (3.2)
1500	68 (1.8)
2000	57 (1.5)
2500	77 (2.0)
3000	62 (1.6)
4000	15 (0.4)

To comply with Danish legislation, we only report numbers >10.
