# Supplementary material for: Prenatal exposure to glucocorticoids and the prevalence of overweight or obesity in childhood
Source: Eur J Endocrinol. 2022 Feb 1;186(4):429–40. doi: 10.1530/EJE-21-0846 (PMC8942335; doi:10.1530/EJE-21-0846)
Supplement: Supplementary Table 4. Definition of covariates. [file supplementary_table_4.pdf]

Supplementary Table 4. Definition of covariates.

	ICD-8 codes	ICD-10 codes	ATC codes
**Potential glucocorticoid treatment indications**			
Obstructive pulmonary disease any time before birth	491, 492, 493	J40- J46	
Inflammatory bowel disease up any time before birth	56301, 56302, 56309, 56319	K50, K51	
Rheumatic disease any time before birth	44630, 44631, 44639, 71219, 71229, 71239, 71259, 69609, 71249, 73400, 73402-4, 73408, 73409, 69549, 73419, 44629, 28709, 44609	M315, M316, M353, M05, M06, M070, M073, L941, M351, M340-M349, M32, G737, N085, N164, M33, M350, M300, M313, D690, M310, I776, L95	
Renal disease any time before birth	24902, 250302, 403, 404, 580-584, 59320, 75311, 75311-19,	N00-08, N11, N14-N16, N18, N19, N26, N27, I12, I13, I150, I151, E102, E112, E142, Q611-Q614	
Skin disease any time before birth	694, 69300, 69308, 69309, 68400	L100, L02, L04, L120, L130, L00, L512, L11, L13, L14	
**Comorbidities**			
Polycystic ovarian syndrome any time before birth	61520, 61521	E282	
Infections or use of antibiotics during pregnancy		O23, A00-A99, B00-B99	J01-J05
Diabetes (type 1, 2 or gestational) any time before birth	249, 250	E10-E14, G632, O24 H360, N083	A10A, A10B
Psychiatric disease any time before birth	290-308	F00-F99	N05, N06A, N06B
Mood or anxiety disorders any time before birth	296, 29809, 300	F30-F48	N06A
Substance use disorders any time before birth	291, 303,304	F10-F19	
Use of antipsychotics during pregnancy			N05A

Abbreviations: ATC: anatomic, therapeutic, chemical. ICD, international classification of diseases.
