# Supplementary material for: Prenatal exposure to glucocorticoids and the prevalence of overweight or obesity in childhood
Source: Eur J Endocrinol. 2022 Feb 1;186(4):429–40. doi: 10.1530/EJE-21-0846 (PMC8942335; doi:10.1530/EJE-21-0846)
Supplement: Supplementary Table 5. Comparison of exposure and baseline characteristics of children with and without anthropometric evaluation at 5-8 years of age. [file supplementary_table_5.pdf]

Supplementary Table 5. Comparison of exposure and baseline characteristics of children with and without anthropometric evaluation at 5-8 years of age.

	Children with anthropometric evaluation at 5-8 years of age	Children without anthropometric evaluation at 5-8 years of age
All births	383,877 (100)	58,401 (100)
Exposure to systemic glucocorticoids	3,883 (1.0)	808 (1.3)
Cumulative systemic glucocorticoid dose in mg prednisolone- equivalents[a], median (IQR)	200 (200-500)	200 (200-375)
Exposure to topical glucocorticoids only	43,131 (11)	5,833 (10)
Male	195,689 (51)	31,183 (53)
Parity, ≥2	203,201 (55)	30,463 (55)
Gestational age, weeks		
< 28	759 (0.20)	309 (0.53)
28 to 31	2,748 (0.72)	514 (0.88)
32 to 36	23,370 (6.1)	3,746 (6.4)
≥ 37	357,000 (93)	53,832 (92)
Birth weight in g, median (IQR)	3,500 (3,150-3,850)	3,460 (3,100-3,810)
SGA	36,418 (9.5)	6,095 (10)
Apgar score after 5 minutes, <7	2,409 (0.63)	1,224 (2.1)
Caesarean section	79,822 (21)	12,158 (21)
Multiple birth	15,980 (4.2)	2,903 (5.0)
Previous spontaneous abortions		
0	319,003 (83)	48,802 (84)
≥1	64,974 (17)	9,599 (16)
Maternal characteristics		
Age at birth (years), median and IQR	30 (27-34)	30 (27-34)
Highest educational level		
Low	61,786 (16)	10,703 (18)
Medium	157,291 (41)	21,561 (37)
High	155,670 (41)	19,886 (34)
Pre-pregnancy body mass index (kg/m^2), median IQR	23 (21-26)	23 (21-27)
<18.5	15,738 (4.1)	2,604 (4.5)
18.5-24	224,757 (59)	32,990 (56)
25-29	75,471 (20)	11,240 (19)
≥ 30	44,578 (12)	6,963 (12)
Smoking during pregnancy, yes	49,296 (13)	7,773 (13)
Obstructive pulmonary disease	16,590 (4.3)	2,458 (4.2)
Inflammatory bowel disease	4,524 (1.2)	660 (1.1)
Rheumatic disease	3,035 (0.79)	418 (0.72)
Renal disease	1,782 (0.46)	305 (0.52)
Skin disease	6,469 (1.7)	986 (1.7)
Diabetes (type I, II, or gestational)	21,199 (5.5)	3,687 (6.3)
Gestational diabetes during pregnancy	11,093 (2.9)	1,879 (3.2)
Infections during pregnancy	113,551 (30)	16,987 (29)
Polycystic ovarian syndrome	4,902 (1.3)	820 (1.4)
Psychiatric illness	97,444 (25)	15,237 (26)

Mood or anxiety disorders	57,374 (15)	9,675 (17)
Substance use disorders	12,205 (3.2)	2,103 (3.6)
Use of antipsychotics during pregnancy	835 (0.22)	193 (0.33)

Abbreviations: IQR, interquartile range. SGA, small for gestational age.
