# Supplementary material for: Prenatal exposure to glucocorticoids and the prevalence of overweight or obesity in childhood
Source: Eur J Endocrinol. 2022 Feb 1;186(4):429–40. doi: 10.1530/EJE-21-0846 (PMC8942335; doi:10.1530/EJE-21-0846)
Supplement: Supplementary Table 6. The association between the baseline characteristics with overweight/obesity in offspring and maternal use of systemic glucocorticoids during pregnancy. [file supplementary_table_6.pdf]

Supplementary Table 6. The association between the baseline characteristics with overweight/obesity in offspring and maternal use of systemic glucocorticoids during pregnancy.

	Prevalence ratio (95% CI) for overweight/obesity at age 5-8 years of age	Prevalence ratio (95% CI) for maternal use of glucocorticoids during pregnancy
Maternal characteristics		
Maternal age at birth, years		
<25	1 (ref)	1 (ref)
25-29	0.75 (0.73-0.77)	1.30 (1.16-1.47)
30-34	0.72 (0.70-0.73)	1.79 (1.60-2.00)
≥35	0.82 (0.79-0.84)	2.80 (2.49-3.15)
Highest educational level		
Low	1 (ref)	1 (ref)
Medium	0.70 (0.69-0.72)	1.00 (0.92-1.11)
High	0.46 (0.45-0.48)	1.23 (1.012-1.36)
Pre-pregnancy body mass index (kg/m^2),		
<25	1 (ref)	1 (ref)
25-29	2.09 (2.05-2.13)	0.91 (0.84-0.99)
≥ 30	3.40 (3.33-3.47)	0.94 (0.85-1.04)
Smoking during pregnancy, yes	1.77 (1.74-1.81)	0.86 (0.77-1.00)
Obstructive pulmonary disease	1.10 (1.06-1.15)	2.85 (2.59-3.14)
Inflammatory bowel disease	1.01 (0.94-1.10)	5.70 (5.02-6.46)
Rheumatic disease	0.99 (0.90-1.09)	7.94 (6.98-9.02)
Renal disease	0.99 (0.88-1.12)	4.28 (3.43-5.35)
Skin disease	1.37 (1.30-1.44)	1.43 (1.17-1.76)
Diabetes (type I, II, or gestational)	1.63 (1.59-1.68)	2.21 (2.00-2.44)
Gestational diabetes during pregnancy	1.82 (1.76-1.89)	2.20 (1.94-2.51)
Infections during pregnancy	1.20 (1.18-1.22)	1.54 (1.45-1.64)
Polycystic ovarian syndrome	1.44 (1.35-1.52)	1.54 (1.23-1.93)
Psychiatric illness	1.16 (1.14-1.19)	1.49 (1.40-1.60)
Mood or anxiety disorders	1.20 (1.18-1.24)	1.37 (1.26-1.48)
Substance use disorders	1.23 (1.18-1.28)	1.13 (0.96-1.34)
Use of antipsychotics during pregnancy	1.66 (1.46-1.90)	2.13 (1.35-3.38)

Abbreviations: CI, confidence interval. Highest educational level at birth: low (primary and lower secondary education), medium (upper secondary education or professional degree) and high (university education at bachelor's degree level or higher
