# Supplementary material for: Prenatal exposure to glucocorticoids and the prevalence of overweight or obesity in childhood
Source: Eur J Endocrinol. 2022 Feb 1;186(4):429–40. doi: 10.1530/EJE-21-0846 (PMC8942335; doi:10.1530/EJE-21-0846)
Supplement: Supplementary Table 7. Sensitivity analysis excluding mothers with potential unmeasured in-hospital glucocorticoid treatment (defined as being hospitalised with a glucocorticoid treatment indication during pregnancy). [file supplementary_table_7.pdf]

Supplementary Table 7. Sensitivity analysis excluding mothers with potential unmeasured in-hospital glucocorticoid treatment (defined as being hospitalised with a glucocorticoid treatment indication during pregnancy).

	Boys (n= 194,233)			Girls (n = 186,766)		
	**Prevalence (%)**	**Crude PR (95% CI)**	**aPR (95% CI)**	**Prevalence (%)**	**Crude PR (95% CI)**	**aPR (95% CI)**
Comparison cohort	11	Ref	Ref	15	Ref	Ref
Exposure to systemic glucocorticoid	10	0.90 (0.77-1.03)	0.90 (0.74-1.00)	14	0.92 (0.82-1.03)	0.94 (0.84-1.06)
1. trimester only	10	0.93 (0.73-1.19)	0.88 (0.68-1.14)	17	1.15 (0.96-1.38)	1.17 (0.98-1.40)
2. trimester only	12	1.31 (0.77-1.72)	1.40 (0.78-1.82)	17	0.92 (0.69-1.22)	0.86 (0.63-1.16)
3. trimester only	9.0	0.80 (0.62-1.01)	0.81 (0.63-1.14)	12	0.76 (0.62-1.02)	0.83 (0.68-1.03)
Multiple trimesters	17	1.62 (1.11-2.34)	1.72 (1.13-2.42)	16	0.91 (0.62-1.32)	0.90 (0.55-1.26)
Cumulative dose						
<250 mg	9.1	0.85 (0.72-1.01)	0.83 (0.68-0.99)	13	0.91 (0.79-1.32)	0.93 (0.80-1.07)
250-499 mg	9.0	0.85 (0.72-1.01)	0.82 (0.68-1.16)	15	1.00 (0.77-1.31)	1.07 (0.82-1.39)
≥ 500 mg	14	1.28 (0.92-1.79)	1.37 (0.90-1.97)	15	1.05 (0.73-1.53)	0.90 (0.82-1.21)
Exposure to topical glucocorticoids only	11	1.02 (0.97-1.06)	0.99 (0.95-1.03)	16	1.08 (0.99-1.12)	1.05 (0.98-1.10)

Adjusted for maternal age at birth (restricted cubic spline with 3 knots), maternal body mass index (BMI) at start of pregnancy (restricted cubic spline with 3 knots), smoking (yes/no), treatment indications, number of hospital contacts with treatment indications within two years prior to birth, maternal type I, II, or gestational diabetes, polycystic ovarian syndrome, psychiatric illnesses, and infections or antibiotic use during pregnancy.
Abbreviations: BMI, body mass index. CI, confidence interval. PR, prevalence ratio.
